# Evaluation of the potential benefits of iron supplementation in organic pig farming

**DOI:** 10.12688/openreseurope.14367.1

**Published:** 2022-01-24

**Authors:** Armelle Prunier, Maud Pauwels, Mily Leblanc-Maridor, Laetitia Jaillardon, Catherine Belloc, Elodie Merlot

**Affiliations:** 1 PEGASE, Inrae, Institut Agro, Saint-Gilles, 35590, France; 2 BIOEPAR, Inrae, Oniris, 44307, France; 3 Laboniris, Oniris, Nantes, 44307, France

**Keywords:** Anaemia, haemoglobin, inflammation, outdoors, indoors, suckling piglet

## Abstract

**Background:** Iron from the stock acquired during foetal life and the ingestion of milk is not sufficient to cover the needs of the piglets during their first weeks of life. In organic farming, systematic supplementation with iron is problematic due to a strong limitation in pharmaceutic treatments.

**Methods:** Erythroid parameters around weaning were measured in piglets from organic outdoor and indoor farms, and related to indicators of the inflammatory status. Blood samples were collected from 28.9±2.6 piglets/herd at 42.0±3.2 days of age and 11.9±3.0 kg live weight (mean ± SD) in 21 farms from the west part of France. Among the 11 outdoor farms, only one had supplemented piglets with 200 mg iron while among the 10 indoor farms, only one had not supplemented piglets, one had supplemented them with 100 mg, 8 with 200 mg and one with 400 mg.

**Results:** Compared to outdoor piglets without supplementation, piglets kept indoors and receiving 200 mg iron had lower haemoglobin concentration (105 vs 118±2 g/l, mean ± SE) and red blood cell volume (56 vs 60±1 fl) (P<0.005). The reduction in haemoglobin concentration and red blood cell volume was more pronounced in indoor piglets supplemented with 100 mg of iron and even more when they had not received iron. The plasma concentration of haptoglobin was lower in outdoor than in indoor piglets (0.51±0.06 vs 0.78±0.09 g/l) whereas no effect of housing was observed for markers of oxidative stress (dROM, BAP). In the 14 farms where sow parity was known, the haemoglobin concentration was lower in piglets from primiparous than from multiparous sows (109 versus 114±2 g/l, P < 0.001).

**Conclusion:** With the exception of soils where the content of bioavailable iron is very low, piglets from outdoor farms do not require iron supplementation, unlike those raised indoors.

## Plain language summary

Iron requirements for haemoglobin synthesis and muscle growth in piglets are very high during their first weeks of life. To meet these requirements, piglets use the iron stored during their foetal life and ingest iron from their mother's milk, but this is not sufficient. In a study supported by an Era-Net CORE Organic project ( Power project), researchers from the French research institute INRAE (Saint-Gilles France) and the veterinary school of Nantes (Oniris, France) explored the iron status of organic piglets raised in free-range or indoor conditions and receiving or not an iron treatment during their first week of life. Such treatment is problematic in organic farming due to a strong limitation of chemically synthetized drugs. About 600 piglets from 21 organic farms in the west part of France were submitted to a blood sample at about six weeks of age, near weaning. The results show signed of anaemia, low haemoglobin content and low red blood cell size in piglets kept indoors receiving a low dose of iron (100 mg) or no iron supplementation. Moreover, the haematologic status was better in outdoor piglets not receiving iron supplementation than in those reared indoors injected with 200 mg dextran or gleptoferron iron. Therefore, except when soils are deficient in bioavailable iron, outdoor piglets do not require iron supplementation unlike those reared indoors. A solution with a continuous supply of iron in a natural form should be developed for organic piglets reared indoors.

## Introduction

The piglet's iron requirement is high during its first weeks of life, about 21 mg per kg of gain, i.e. 5 to 7 mg per day, to accompany its rapid growth ( Braude *et al.*, 1962; Mahan & Shields, 1998; Venn *et al.*, 1947). Iron is used, in particular, for the synthesis of haemoglobin in red blood cells and myoglobin in muscle. To meet the iron requirement, the new born piglet mobilizes its reserves stored during foetal life, but they only cover a few days of life ( Mahan *et al.*, 2009; Venn *et al.*, 1947). A second source of iron is maternal milk, but the intake of about 1 mg per day ( Szudzik *et al.*, 2018; Venn *et al.*, 1947) is very low compared to the need. Piglets would therefore become anaemic very quickly if they do not have access to other sources of iron. When lactating sows are kept outdoors, the absence of iron supplementation does not seem to be a problem, probably because piglets ingest iron from the soil ( Brown *et al.*, 1996; Delbor *et al.*, 2000). When sows are kept indoors, this ingestion of soil does not occur and iron supplementation is necessary ( Svoboda *et al.*, 2017; Svoboda & Pist'kova, 2018).

In conventional sows, farmers bring iron to piglets in the first few days of life by parenteral (intramuscular injection) or oral supplementation ( Svoboda *et al.*, 2017; Svoboda & Pist'kova, 2018; Szudzik *et al.*, 2018). In organic farming this systematic supplementation is problematic since it breaks the principles of health (i.e. animal drugs should be avoided) and ecology (i.e. the production is to be based on ecological processes) ( IFOAM, 2020). In accordance with these principles, the EU regulation for organic livestock farming limits strongly the use of chemically synthesised drugs ( Commission Regulation EC n° 889/2008, 2008). For a pig to be certified as organic, only one drug treatment is allowed apart from parasite treatments, vaccines and compulsory treatments (e.g. mitigation of pain derived from male castration). Even if not systematic, iron preparations administered to piglets are considered, by some certifying bodies, to be a drug treatment. In this case, the meat of a pig will be downgraded if it receives another treatment to treat a disease. This leads some farmers not to perform neonatal iron injections.

As little data are available on the risk of iron deficiency in organic pig farms, the main objective of this study was to measure haematological parameters as well as circulating concentrations of iron and ferritin in organic piglets raised outdoors or indoors and born from primiparous or multiparous mothers. Their inflammatory state was also assessed by measuring an inflammation protein (haptoglobin) and oxidative stress indicators because the inflammatory status can modulate the availability of iron for haemoglobin synthesis ( Ganz & Nemeth, 2012).

## Methods

### Animals and sampling

All procedures were approved by the ethical committee for Clinical and Epidemiological Research from the Oniris Veterinarian School of Nantes in France (CERVO) and received the agreement # CERVO-2018–15-V. All efforts were made to minimise animal suffering. Farmers enrolled in the study were informed about the aims and content the project and signed a letter of informed consent.

Twenty-one organic pig farms (11 outdoors, 10 indoors), located in the west part of France, were selected on a voluntary basis. Domestic pigs (Sus scrofa domesticus) were selected within litters so that males and females, low, medium and large piglets were equally represented. In addition, only males with both testes descended were selected. In each farm, 28.9 ± 2.6 [18, 30] piglets (average ± SD, [minimum, maximum]) from four to seven litters (sows of different parities) were sampled at 42.0 ± 3.2 [34, 57] days of age, 11.9 ± 3.0 [5, 23] kg live weight and -1.1 ± 1.6 [-6, +1] days from weaning (606 pigs in total). Live weight was measured individually with a portable scale (HDB 30 K-2XL, 2336 Balingen, Germany). Blood was drawn from the jugular vein to collect 5 ml on a dry tube and 5 ml on an EDTA tube. Each piglet was kindly caught by the farmer or a technician. It was firmly held on its back in order to prevent any movement and to have the forelegs spread. The second operator, previously trained to blood sampling in piglets, performed the sampling. Immediately after, the piglet was weighed and returned to its home pen. The whole procedure lasted less than two minutes. The samples were stored, for a maximum of four hours, in a refrigerated cooler until arrival at the laboratory.

As far as possible, the identity of the mother, parity and date of birth were recorded. In order to link the piglets with their mother in outdoor farms, farmers were required to fix an identification tag on the ear of the piglets shortly after birth. When the exact date of birth was not known, a single date was recorded according to the farmer indication.

### Laboratory analyses

On arrival at the laboratory, a complete haematological analysis was carried out on the blood collected on EDTA using a Procyte Dx Idexx automaton (Westbrook, Maine 04092, USA). Parameters measured included haemoglobin concentration (Hb), haematocrit, red blood cell and reticulocyte counts, mean Red Blood Cell Volume (RBCV), the mean Red Blood Cell Haemoglobin Concentration (RBCHb), the Reticulocyte Haemoglobin concentration (RetHb), the number of lymphocytes, monocytes, neutrophilic and eosinophilic granulocytes. These analyses were performed on the 606 piglets for all parameters except the leucocyte count for which three samples could not be analysed for technical reasons.

The remaining blood on EDTA was centrifuged before collecting the plasma. After clotting, the dry blood was centrifuged and the serum was collected. The aliquots were stored, for a maximum of four months, at -20°C until analysis. Haptoglobin was analysed on plasma, iron, ferritin, hydroperoxides (= oxidation products = dROM) and the antioxidant capacity of the blood (BAP) on serum. All analyses except for ferritin were performed in single assays with a laboratory robot (Konelab 20, Thermo Scientific, Waltham, MA USA) using commercial kits that are not species specific (total iron: Iron- 981236, Thermo Scientific, Finland; haptoglobin: T801, Tridelta Ltd, Maynooth, Ireland; dROM and BAP: Diacron, Grosseto, Italy). The intra-assay CV was 5.5%, 7%, 4% and 8%, respectively for iron, haptoglobin, dROM and BAP. Ferritin was measured in duplicates using an ELISA test specific for pigs (kit MBS741259, MyBioSource, San Diego, CA 92195–3308). The intra-assay CV was 3%.

These analyses were performed on 552, 566, 565, 563 piglets (18 to 30 piglets per farm), for iron, BAP, haptoglobin and dROM respectively, as they were not carried out in one outdoor farm (Farm U) and on a few pigs in eight other farms due to a lack of plasma or to a technical problem. Ferritin was measured in 200 piglets (10 piglets per farm selected to ensure a balanced sex ratio and diversity of weights within farms) from all farms except one outdoor farm (farm U).

### Statistical analyses

All analyses were performed with the R software (version 4.0.2). Mixed ANOVA (type 3) were carried using the lmerTest and Car packages. To analyse live weight and daily weight gain (DWG), sex (two modalities: Male vs Female) and housing (two modalities: Indoors vs Outdoors) were introduced as fixed effects and farm as a random effect. To analyse blood parameters, sex (two modalities: Male vs Female), housing (two modalities: Indoors vs Outdoors), live weight (quantitative variable) were introduced as fixed effects and farm as a random effect. In a subgroup of 13 farms where sow parity was known and piglets clearly linked to their dam, parity (two modalities: Primiparous vs Multiparous) was also included. A square root transformation was carried out before analysis for some parameters (number of lymphocytes, monocytes, neutrophils and eosinophils, iron, ferritin, haptoglobin and dROM concentrations, BAP) to normalise them and homogenise the variances. Pearson correlations between variables were calculated with the Hmisc package. Statistical levels were corrected for multiple comparison using the Holm method. Principal Component Analyses were performed with the FactoMineR package and missing values were imputed by the mean of each variable. Unless otherwise stated, the results presented in the text correspond to adjusted means ± SEM. P-values < 0.05 were considered statistically significant.

## Results

**Figure 1.**
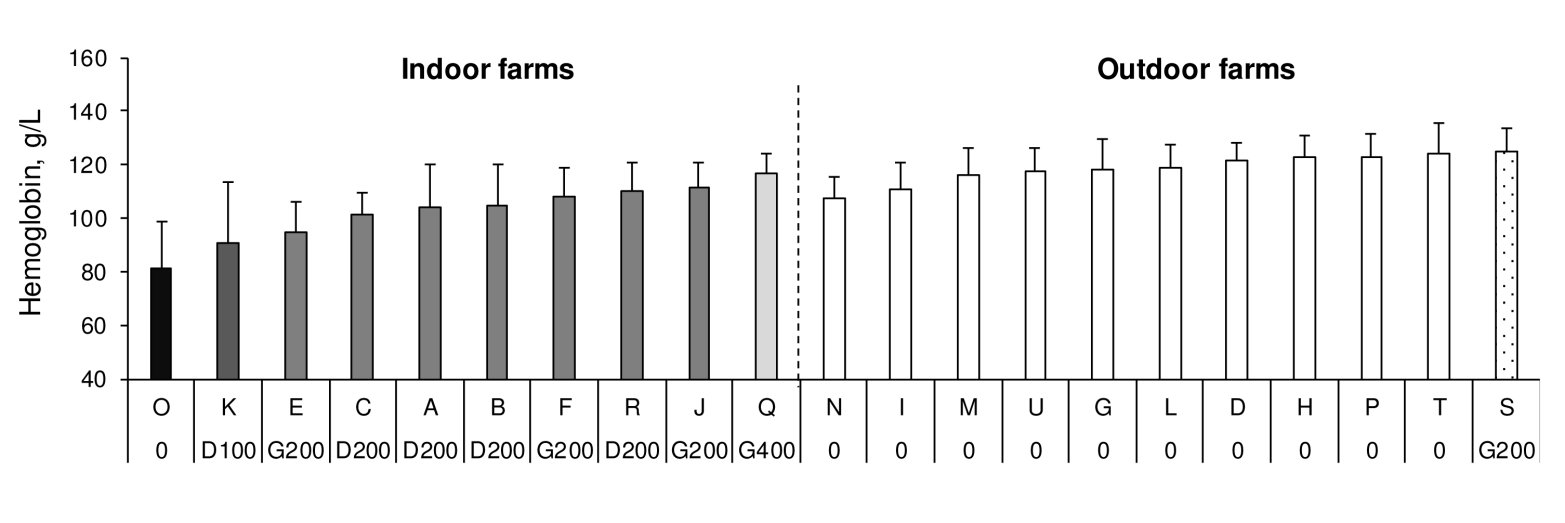
Mean blood haemoglobin concentration of piglets in 10 indoor and 11 outdoor organic farms. Blood haemoglobin concentration in the 21 farms enrolled in the study (mean ± SD). 0: no iron injection, D100: injection of 100 mg iron dextran, D200: injection of 200 mg iron dextran, G200: injection of 200 mg gleptoferron iron, G 400: injection of 400 mg gleptoferron iron.

In only one indoor farm (O), no iron supplementation was carried out. In the other indoor farms, piglets received: 100 (K) or 200 (A, B, C and R) mg dextran iron, 200 (E, F and J) or 400 (Q) mg gleptoferron iron ( Figure 1). The injection was performed between one and four days of age except in the Q (on the day of birth) and C (four days of age) farms. Irrespective of the nature of the iron injected, descriptive statistics show that, indoors, the average haemoglobin concentration progressively increases between 0 (81.0 g/l), 100 (90.5 g/l) and 200 mg of iron (> 94.5 g/l), and between 200 (108.1 to 111.3 g/l) and 400 mg (116.7 g/l) of iron. In the outdoor farms, the piglets did not receive any iron supplementation except in one farm (S) where 200 mg gleptoferron iron was injected at seven days of age ( Figure 1). In this farm, mean haemoglobin concentration was very close (124.8 g/l) to the mean measured in the four highest farms not injecting iron (121.2 to 124.6 g/l).

No statistical analysis was carried out to test the influence of the iron dose because only one farm injected 0, 100 or 400 mg of iron indoors and 200 mg of iron outdoors. Similarly, the number of farms was insufficient to statistically compare the two forms of iron when 200 mg iron was supplied.

### Effects of housing, sex and live weight of piglets

Farms O, K, Q and S were excluded from the statistical analyses for the comparison of housing because of their particular practice of iron supplementation (no or 100 mg injection indoors, 200 mg outdoors, see above). Indoor farms supplementing with 200 mg iron using dextran or gleptoferron forms were grouped together in the same category. Finally, 7 indoor (206 piglets) and 10 outdoor (280 piglets) farms were compared.

Live weight (Indoors: 11.4 ± 0.6 kg, Outdoors: 12.7 ± 0.5 kg; P > 0.1), DWG (Indoors: 0.28 ± 0.01 kg/day, Outdoors: 0.29 ± 0.01 kg/day; P > 0.1) and age of the piglets (Indoors: 40.8 ± 0.2 days, Outdoors: 43.3 ± 0.2 days; P < 0.001) at sampling were close in both housing systems even if a significant difference was observed for the age.

### Parameters of the erythrocyte lineage, serum iron and ferritin

**Figure 2.**
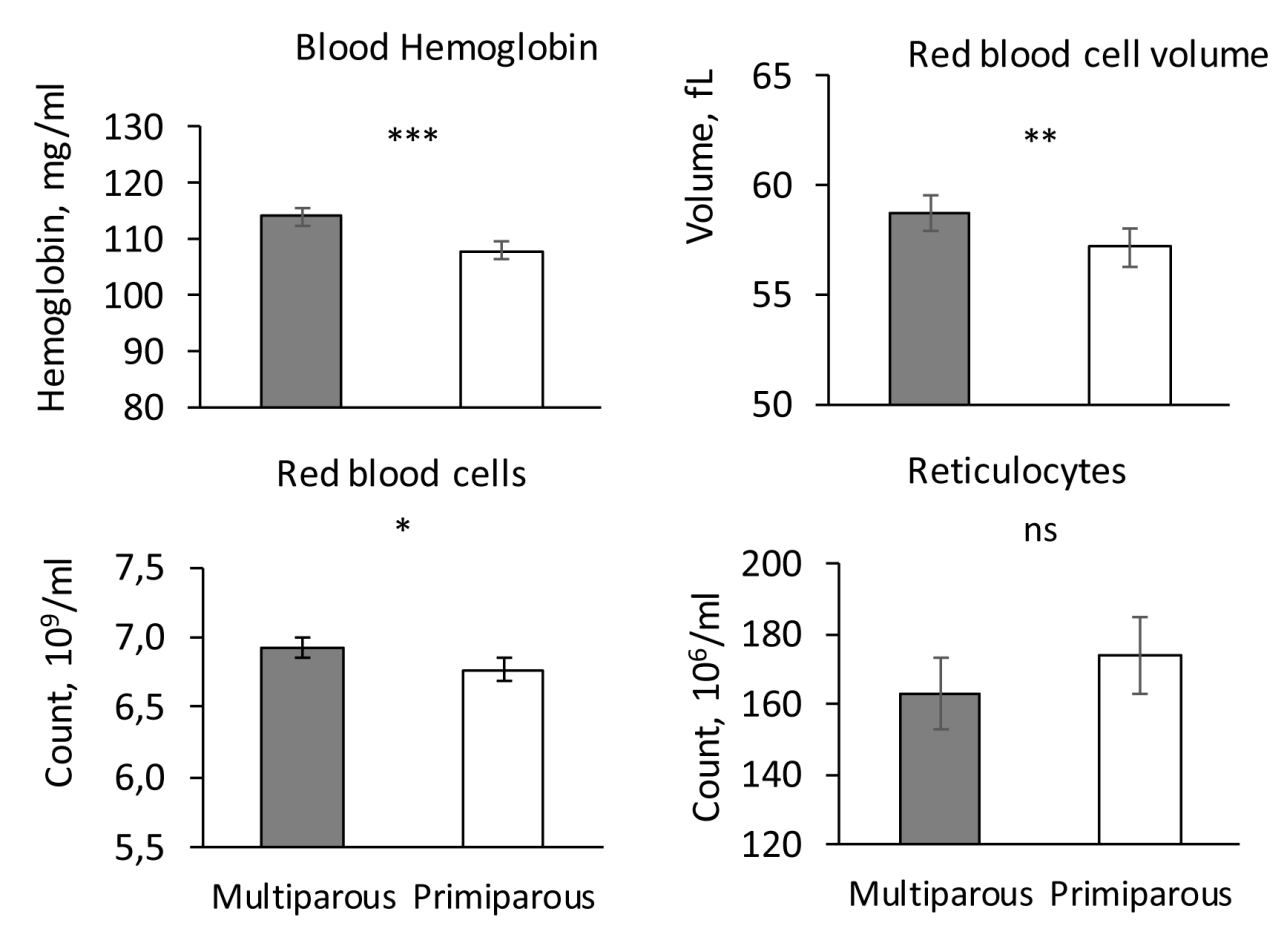
Haematologic indicators in indoor (200 mg iron supply) and outdoor piglets (no iron supply). Influence of housing (Indoors vs. Outdoors) on blood haemoglobin concentration, Reb Blood Cell Volume (RBCV), red blood cell and reticulocyte counts (adjusted mean ± SE). *** P < 0.001, ** P < 0.01, * P < 00.05, ns P > 0.1.

Haemoglobin concentration, haematocrit, red blood cell count, RBCV, RBCHb and RetHb were significantly higher in piglets kept outdoors compared to piglets kept indoors (P < 0.05, Figure 2). The reticulocyte count, the concentrations of iron and ferritin did not differ significantly between the two housing systems (P > 0.1).

There was a significant effect of sex only for RetHb and blood haemoglobin concentration, both parameters being significantly lower in males (haemoglobin: 110.0 ± 1.5 mg/ml; RetHb: 17.0 ± 0.2 µg/ml) than in females (haemoglobin: 113.0 ± 1.5 mg/ml; RetHb: 17.4 ± 0.2 10 µg/ml; P < 0.05). Live weight had a significant influence only on the reticulocyte count which increased with weight (P < 0.003).

### Number of leukocytes and inflammatory status

**Figure 3.**
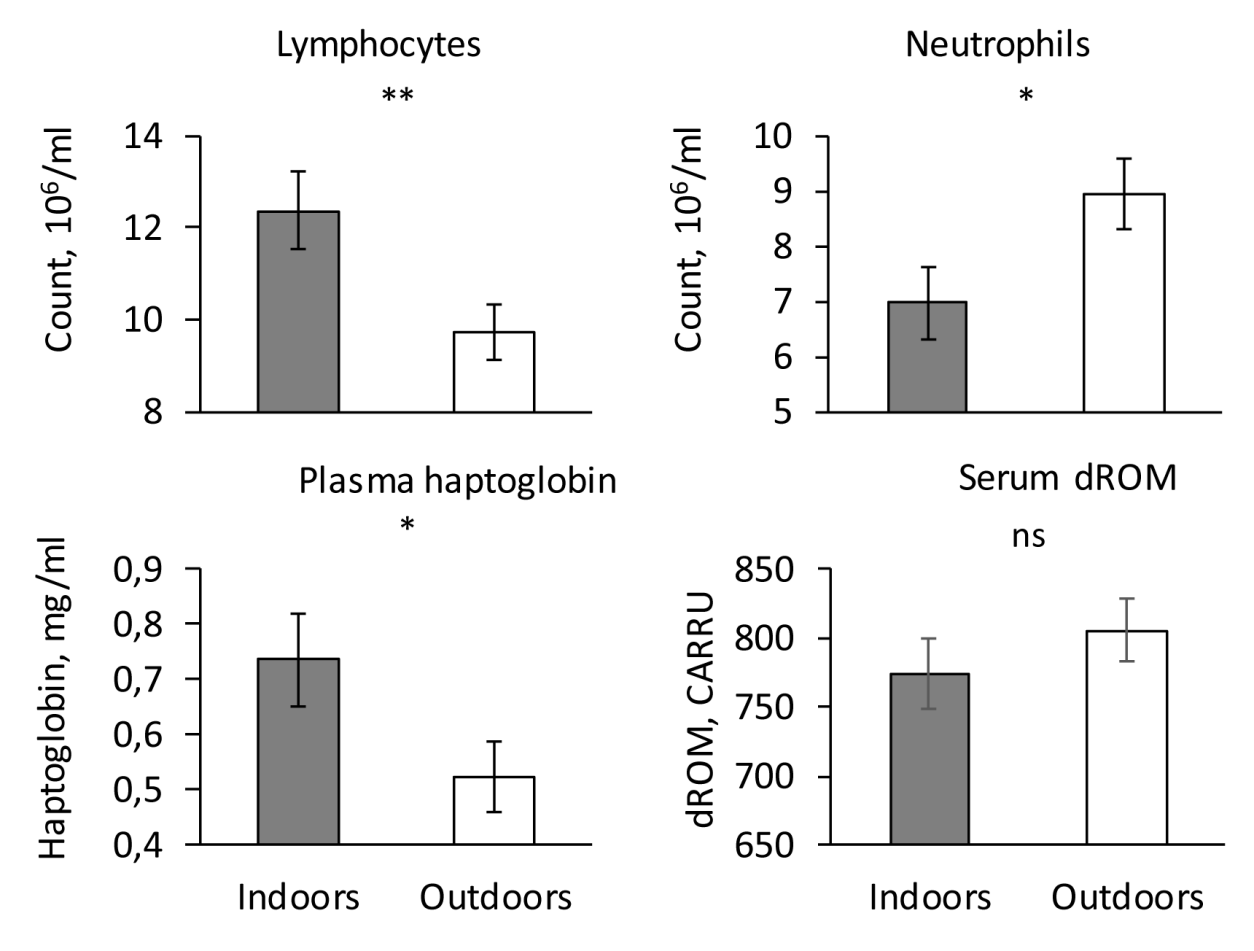
Health indicators in indoor (200 mg iron supply) and outdoor piglets (no iron supply). Influence of housing (Indoors vs. Outdoors) on lymphocyte and neutrophil counts, plasma haptoglobin concentration and serum dROM concentration (adjusted mean ± SE). ** P < 0.01, * P < 0.05, ns P > 0.1.

The number of lymphocytes was significantly higher and the number of neutrophils lower indoors than outdoors (P < 0.05, Figure 3). The numbers of monocytes and eosinophils did not differ significantly between the two housing systems (P > 0.1). Serum haptoglobin concentration was higher in indoor than outdoor piglets (P < 0.05, Figure 3) whereas serum dROM and BAP were similar in both housing systems (P > 0.1, Figure 3).

A significant sex effect was only observed for neutrophils with a higher number in males than in females (8.21 ± 0.50 vs. 7.65 ± 0.48 106 cells/ml). Body weight had a significant effect only on the number of lymphocytes (P < 0.003), BAP (P <0.002), serum haptoglobin (P < 0.007) and dROM (P < 0.02). The number of lymphocytes and BAP increased with increasing body weight whereas serum haptoglobin and dROM decreased with increasing body weight.

### Effects of parity

The comparison between primiparous and multiparous sows was only carried out in farms where parity was known and where at least two piglets of each sex and each type of sow had been measured. A total of five indoor and eight outdoor farms were included with 122 piglets from primiparous sows and 258 from multiparous sows (parities 2 to 9).

### Parameters of the erythrocyte lineage, serum iron and ferritin

**Figure 4.**
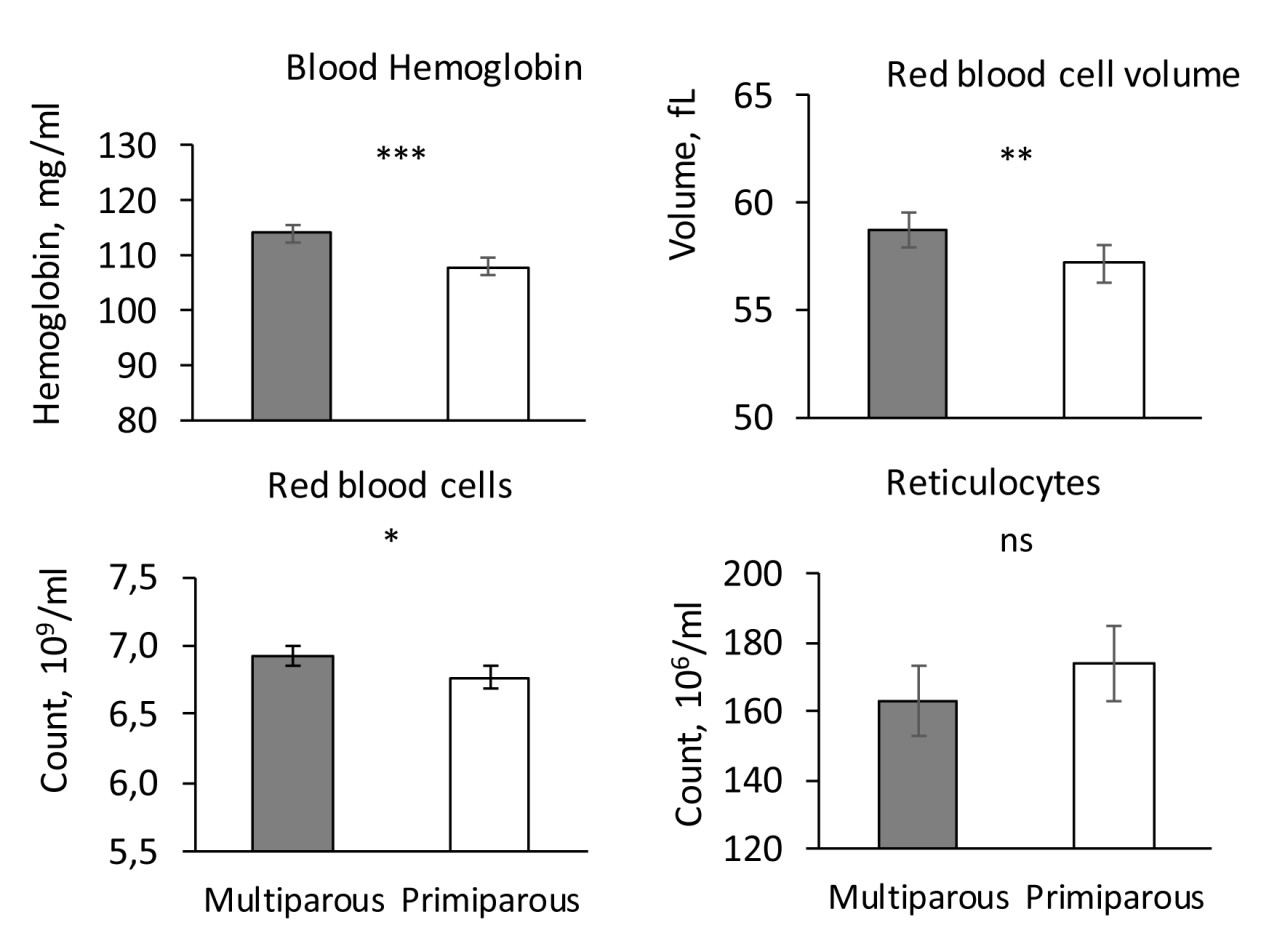
Haematologic in piglets from primiparous and multiparous sows. Influence of sow parity (Primiparous vs. Multiparous) on blood haemoglobin concentration, Red Blood Cell Volume (RBCV), red blood cell and reticulocyte counts (adjusted mean ± SE). *** P < 0.001, ** P < 0.01, * P < 00.05, ns P > 0.1.

The influence of parity was significant for haemoglobin concentration, red blood cell count, RBCV (P < 0.05, Figure 4), haematocrit (Multiparous: 40.6 ± 0.7 %, Primiparous: 38.6 ± 0.7%, P < 0.05) and ferritin (Multiparous: 15.7 ± 1.1 pg/ml, Primiparous: 19.3 ± 1.1 ng/ml, P < 0.05) but not for RBCHb, reticulocyte count and RetHb (P > 0.1, data not shown).

### Number of leukocytes and inflammatory status

Only the number of monocytes was significantly influenced by sow parity, piglets from primiparous sows (1.49 ± 0.11 10 ^6^ cells/ml) having more monocytes than those from multiparous sows (1.31 ± 0.10 10 ^6^ cells/ml; P > 0.004).

### Relationships between parameters

Correlation analyses were carried out separately for the 7 indoor farms injecting 200 mg iron and for the 10 outdoor farms performing no supplementation. Only five correlations were consistent in both housing systems: positive between blood Hb on one side and RBCV or red blood cell count on the other side (P < 0.001 for both correlations and systems) as well as between plasma haptoglobin and serum dROM (P < 0.001 for both systems) but negative between serum haptoglobin on one side and the reticulocyte count (Outdoors: P < 0.03, Indoors: P < 0.001) or serum iron (Outdoors: P < 0.07, Indoors: P < 0.001) on the other side ( Table 1). One correlation was opposite in the two housing systems, that between plasma haemoglobin and average daily gain (Outdoors: positive and P < 0.07, Indoors: negative and P < 0.03, Table 1). Four correlations were significant only Outdoors and sixteen only Indoors ( Table 1). The most interesting ones were positive correlations between serum iron on one side and plasma Hb or RBCV on the other side in Indoor pigs (P < 0.001 for both correlations, Table 1).

**Table 1.** Correlations between haematologic and health indicators in outdoor and indoor pigs. Pearson correlations between blood (haemoglobin concentration: Hb, red blood cell volume: RBCV, red blood cell count: nRBC, reticulocyte count: nRetic, square root of neutrophil count: rnNeutro, square root of lymphocyte: rnLympho), serum (square root of iron concentration: rIron, square root of hydroperoxide concentration: rdROM, antioxidant capacity: BAP) and plasma (square root of haptoglobin concentration (rHapto) characteristics, and daily weight gain (DWG). Values above the diagonal are calculated for Outdoor pigs (n = 242 to 280) and values below the diagonal are calculated for Indoor pigs (n = 188 to 203). Bold characters indicate that the correlation is significant (P < 0.5). Bold and Italic characters indicate that the correlation tends to be significant (P < 0.1). Levels of significance were corrected for multiple comparisons using the Holm method.

		**Outdoors**										
		Hb	RBCV	nRBC	nRetic	rnNeutro	rnLympho	rIron	rHapto	rdROM	BAP	DWG
**Indoors**	Hb	1.00	**0.53**	**0.75**	0.00	-0.09	0.07	0.10	-0.10	-0.05	0.15	***0.19***
	RBCV	**0.59**	1.00	-0.03	0.09	**-0.22**	0.18	-0.01	-0.15	-0.06	0.05	***0.18***
	nRBC	**0.48**	**-0.37**	1.00	-0.04	0.04	0.05	0.10	0.05	0.00	**0.22**	0.09
	nRetic	**0.27**	**0.48**	-0.11	1.00	0.03	0.11	0.15	**-0.22**	0.01	0.18	0.11
	rnNeutro	0.13	-0.09	**0.25**	-0.15	1.00	0.12	**-0.24**	0.06	0.02	0.04	0.09
	rnLympho	0.14	-0.14	**0.38**	0.02	0.17	1.00	0.01	-0.09	0.04	0.08	0.17
	rIron	**0.57**	**0.52**	0.05	**0.32**	-0.04	0.10	1.00	***-0.20***	-0.07	0.04	-0.06
	rHapto	**-0.29**	**-0.36**	0.04	**-0.31**	**0.29**	-0.18	**-0.41**	1.00	**0.35**	-0.10	***-0.20***
	rdROM	0.09	0.16	-0.05	-0.10	**0.26**	-0.12	0.00	**0.48**	1.00	**0.31**	-0.07
	BAP	-0.13	0.07	-0.13	0.10	**-0.23**	-0.03	-0.14	-0.07	0.10	1.00	0.12
	DWG	**-0.23**	-0.17	0.02	**0.27**	**-0.22**	0.02	-0.16	-0.13	**-0.30**	**0.25**	1.00

**Figure 5.**
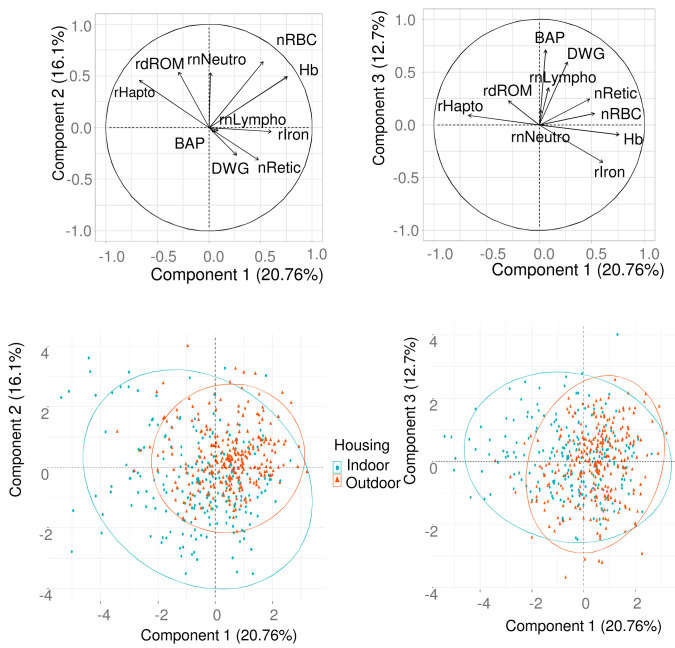
Representation of the links between haematologic and health indicators and projection of the piglets. Overall pattern of correlations between blood (haemoglobin concentration: Hb, reticulocyte count: nRetic, square root of neutrophil count: rnNeutro, square root of lymphocyte: rnLympho), serum (square root of iron concentration: rIron, square root of hydroperoxide concentration: rdROM, antioxidant capacity: BAP) and plasma (square root of haptoglobin concentration (rHapto) characteristics, and daily weight gain (DWG), presented on the first (first and second components, A) and second (first and third components, B) plots. Projection of the piglets on to the first (first and second components, C) and second (first and third components, D) plots. Ellipses include 95% of the individuals.

Multivariate analysis (PCA) was performed across housing systems using the 486 piglets from the same 7 indoor and 10 outdoor farms. Three traits related to the haematologic status (blood Hb, red blood cell and reticulocyte counts), five traits related to the health and oxidant status (neutrophil and lymphocyte counts, plasma haptoglobin, serum dROM and BAP), average daily gain and serum iron were included. Red blood cell volume was not included since it was highly correlated to the reticulocyte count. The first three components of the PCA explained respectively 20.8, 16.1 and 12.7%, i.e. a total of 49.5% of the total variance. The highest contributors to the first component were blood haemoglobin, plasma haptoglobin, serum iron and red blood cell count in decreasing order. The highest contributors to the second component were red blood cell count, neutrophil count and dROM in decreasing order. The highest contributors to the third component were serum BAP and daily weight gain in decreasing order. The correlation structure between the 10 traits showed that (a) plasma haptoglobin was opposite to serum iron, blood haemoglobin and blood cell count, (b) serum iron, blood haemoglobin and blood cell count were positively linked, (c) BAP and daily weight gain were positively linked ( Figure 5A and 5B). Projection of the piglets on to the first ( Figure 5C) and second ( Figure 5D) plots showed that indoor piglets were much more dispersed than outdoor ones. The difference between housing systems was essentially related to the projection on the first component with some indoor piglets characterized by high plasma haptoglobin, low serum iron, blood haemoglobin and blood cell count.

## Discussion

This study assessed the possible consequences of different iron intakes on the haematologic status and the inflammatory state of piglets reared in organic pig farms either indoors or outdoors. They show that iron supplementation is not necessary in the open air, in line with most previous results ( Brown *et al.*, 1996; Delbor *et al.*, 2000; Kleinbeck & McGlone, 1999). It is most likely the supply of iron through the consumption of soil that explains why piglets reared outdoors do not suffer from anaemia even if they do not receive iron supplementation ( Venn *et al.*, 1947). In support of this, a three-week-old piglet reared outdoors was shown to have 513 mg of iron in its digestive contents ( Venn *et al.*, 1947). However, the risk of anaemia exists when soils have a very low content of bioavailable iron. Indeed, Brown *et al.* (1996) showed an abnormally low average haemoglobin concentration at 3–4 weeks of age in one farm among the eight investigated in their study in Scotland (84 mg/ml compared with 103–124 mg/ml), and this farm was the one with the lowest iron content in its soil. More markedly, Szabo & Bilkei (2002) showed an extremely low mean haemoglobin concentration at five weeks of age (54.1 mg/ml) in a Hungarian outdoor farm where piglets did not receive iron supplementation.

On the other hand, indoors, a dose of 200 mg of iron per piglet is a minimum to reach a sufficient level of haemoglobin. Without the iron injection or with a supplementation of 100 mg, the haemoglobin concentration measured in the indoor farms of the present study was very low. This is in line with the bibliography which shows that 100 mg are clearly too low whereas 200 mg of iron would cover the iron requirement up to three weeks of age but would be insufficient beyond that, unless the piglets have access to an iron source complementary to milk ( Svoboda *et al.*, 2017). Indeed, at three weeks of age, piglets would have consumed their prenatal reserve and the stock provided by the 200 mg neonatal supplementation, due to the very deficient balance between the high need for rapid growth and the relatively low intake from milk ( Svoboda *et al.*, 2017).

Our study shows that the iron status of outdoor piglets is better than that of indoor piglets receiving an iron injection of 200 mg. All measured parameters were in the same direction. Haemoglobin concentration, haematocrit, red blood cell count, mean corpuscular haemoglobin concentration, mean reticulocyte haemoglobin concentration, and red blood cell volume were lower in piglets raised indoors, suggesting an iron deficiency in those piglets compared to outdoor piglets. A priori, the iron intake from milk and solid feed did not differ between the two systems since piglets had comparable body weight and average daily gain. Thus, two hypotheses may explain this iron deficit in indoor pigs: iron sequestration due to a more pronounced inflammatory state or insufficient iron intake. Long lasting inflammation favours anaemia because it triggers the secretion of hepcidin, an hormone that reduces iron bioavailability by enhancing its sequestration in macrophages ( Hentze *et al.*, 2010). The more pronounced inflammatory state indoors is suggested by a significantly higher plasma haptoglobin concentration even though the lack of effect of the environment on oxidative stress indicators suggests that the severity of this inflammation was limited. A significant correlation was also identified between plasma haptoglobin and serum iron, which was more marked indoors than outdoors. This supports that high haptoglobin may have contributed to an anaemic status of indoor piglets.

The more marked inflammatory state of piglets kept indoors reveals a higher activity of their immune system. We also observed more lymphocytes but fewer neutrophils indoors than outdoors in agreement with previous results ( Kleinbeck & McGlone, 1999). The origin of the inflammatory response and change in leucocyte counts was not determined as we did not measure any clinical parameters and data from the literature can hardly be used. Indeed, to our knowledge, there is no study comparing the health of organic piglets raised outdoors and indoors. However, studies performed in older pigs suggest that there are more digestive and respiratory health problems indoors than outdoors ( Delsart *et al.*, 2020; Leeb *et al.*, 2019).

The second hypothesis is that iron intake, through soil licking or consumption, allowed an adequate iron intake throughout lactation in outdoor pigs whereas in indoor pigs pre and post-natal stock were probably depleted by three weeks of age (see above) and intake by milk and feed were not sufficient to meet the need (see above). The iron intake from soil in outdoor piglets would have "compensated" or even be higher than that due to the neonatal injection of 200 mg of iron in indoor pigs. This superiority of iron intake via the soil can be understood in terms of quantity but also in terms of quality. Indeed, the neonatal injection of a massive dose of iron is likely to induce oxidative stress and to promote inflammatory states, such as arthritis ( Svoboda *et al.*, 2017; Szudzik *et al.*, 2018) that may in turn favour the sequestration of iron. In addition, iron dextran injection was shown to induce a high expression of hepcidin and a low expression of ferroportin in the duodenum ( Pu *et al.*, 2015) that may reduce iron absorption from milk and feed. The supply of iron from the earth is obviously very gradual and probably adapted to the needs of animals whose ancestral way of life, during which the species has evolved the longest, allowed the consumption of land.

A positive correlation between serum iron and blood haemoglobin was observed in indoor pigs (r = 0.57) in agreement with the literature in Human ( Hinrichs *et al.*, 2010; Mahiou *et al.*, 1992) or cattle ( Joerling & Doll, 2019). However, such a high correlation was not present in outdoor pigs (r =0.1) suggesting that the regulation of iron storage and the use of iron for haemoglobin synthesis were somewhat different in indoor and outdoor pigs. Interestingly, the results from the multivariate analysis indicated a greater heterogeneity regarding immune, inflammatory and red blood cell indicators among piglets raised indoors. This supports the hypothesis that piglets raised indoors are more at risk of developing anaemia and a health disorder whatever the cause.

Our results indicate a poorer haematologic status in piglets from primiparous sows than from multiparous sows, with a lower blood haemoglobin concentration and a lower average globular volume. This result cannot be compared with the bibliography as, to our best of knowledge, this is the first study to make this type of comparison. The difference could be explained by the fact that primiparous sows have not finished their growth so that the export of iron to the foetus or milk would compete with the sows' need for their own growth. Measurements in conventionally reared sows have shown a higher haemoglobin concentration in primiparous sows than in multiparous sows at different stages of gestation and lactation in France ( Normand *et al.*, 2012) and the USA ( Castevens *et al.*, 2020) and hence suggested iron deficiency in multiparous sows that may lead to iron deficiency in their progeny at the opposite of present results. However, they did not measure the iron stock at birth or the blood haemoglobin concentration in lactating piglets in the progeny to prove their hypothesis. In addition, it should be mentioned that these studies were carried out in conventional farms where lactation is significantly shorter (around four weeks in France and three weeks in the USA) than in our study (around six weeks) so that sows would have much less time to recover from iron transfer to their foetuses during gestation and blood loss at farrowing. This would result in a depletion of iron reserves over parities and, probably, low transfer to their progeny in conventional sows which would not occur in organic farming.

## Conclusion

This study shows that piglets reared outdoors find sufficient iron in their natural environment and do not need iron supplementation. Indoors, supplementation is necessary, but a single intramuscular injection of 200 mg of iron may be sub-optimal to prevent anaemia. Moreover, as this injection can be considered as a drug treatment in organic pig farming, alternative solutions need to be found to ensure a sufficient, natural and progressive iron supply to piglets kept indoors.

## Data availability

### Underlying data

Data INRAE: Iron supplementation in organic pig farming, https://doi.org/10.15454/QWRJHL.

This project contains the following underlying data:

- DataSetFinal_Power_21OrganicFarms.tab
- Variable_List_DataSetFinal_Power1.tab

Data are available under the terms of the Creative Commons Zero "No rights reserved" data waiver (CC0 1.0 Public domain dedication).
