# Supplementary material for: Evaluation of the potential benefits of iron supplementation in organic pig farming
Source: Open Res Eur. 2022 Mar 21;2:11. Originally published 2022 Jan 24. [Version 2] doi: 10.12688/openreseurope.14367.2 (PMC10445923; doi:10.12688/openreseurope.14367.2)
Supplement: Peer review history

# Peer review: Reviewer response for version 2

**DOI:** 10.21956/openreseurope.15798.r28889

**Published:** 2022-03-25

**Authors:** Glen Almond

**Affiliations:** 1 Department of Population Health & Pathobiology, College of Veterinary Medicine, North Carolina State University (NCSU), Raleigh, NC, USA

The authors addressed this reviewer's concerns. It is challenging to control or measure all factors in an applied study over 21 farms. The information should prove useful. Field studies are challenging; however, the information is readily applied to commercial farms. Well done!

Is the study design appropriate and does the work have academic merit?

Yes

Is the work clearly and accurately presented and does it cite the current literature?

Partly

If applicable, is the statistical analysis and its interpretation appropriate?

I cannot comment. A qualified statistician is required.

Are all the source data underlying the results available to ensure full reproducibility?

Partly

Are the conclusions drawn adequately supported by the results?

Yes

Are sufficient details of methods and analysis provided to allow replication by others?

Partly

Reviewer Expertise:

40 years of basic and applied research in commercial pig production. Current research is directed at evaluating anemia in commercial pig farms. Previous research studies involved UTI's in sows and non-infectious reproductive failure in pigs.

I confirm that I have read this submission and believe that I have an appropriate level of expertise to confirm that it is of an acceptable scientific standard.

# Peer review: Reviewer response for version 1

**DOI:** 10.21956/openreseurope.15505.r28451

**Published:** 2022-02-08

**Authors:** Martin Svoboda

**Affiliations:** 1 Faculty of Veterinary Medicine, Ruminant and Swine Clinic, University of Veterinary and Pharmaceutical Sciences Brno, Brno, Czech Republic

The article deals with a very important topic. Iron deficiency anaemia is a serious problem in swine production, it can cause significant economic losses. Therefore it is very important to find an optimal way of iron supplementation. The paper is very well written and also its technical quality is very good.

 I recommend it pass peer review, but I have the following recommendations for any future versions:

- Please add information about the creep feet I mean iron content and from which day it was offered to the piglets.
- Please add information about the weaning age of piglets.
- Please give information about the breeds of pigs in different farms.
- I think that the limitation of the study is that the blood was taken only at the weaning and not for example in the first two weeks of life because with voluntary consumption I would expect differences in the first weeks of life of piglets. In my experience with voluntary consumption of iron, there is a usually high variability among piglets in the first weeks of life. So take this only as a recommendation for future studies.

Is the study design appropriate and does the work have academic merit?

Yes

Is the work clearly and accurately presented and does it cite the current literature?

Yes

If applicable, is the statistical analysis and its interpretation appropriate?

Yes

Are all the source data underlying the results available to ensure full reproducibility?

Yes

Are the conclusions drawn adequately supported by the results?

Yes

Are sufficient details of methods and analysis provided to allow replication by others?

Yes

Reviewer Expertise:

NA

I confirm that I have read this submission and believe that I have an appropriate level of expertise to confirm that it is of an acceptable scientific standard.

# Response

**Published:** 2022-03-11

**Authors:** armelle prunier

**Affiliations:** inrae, France

Thanks a lot for your valuable evaluation and comments.

- Please add information about the creep feet I mean iron content and from which day it was offered to the piglets.

It is an on-farm experiment and we do not have a precise information on that. We have added a table (Table 1) to describe this information we got during the farm visits together with some other characteristics of the farms as requested in other comments. We also added some comments in the text.

- Please add information about the weaning age of piglets.

Age at sampling and days from weaning were included in the text. Therefore, age at weaning could be deduced from that. However, we have added this information in Table 1 and added some comments in the text.

- Please give information about the breeds of pigs in different farms.

We have added this information in Table 1 and in added some comments in the text.

- I think that the limitation of the study is that the blood was taken only at the weaning and not for example in the first two weeks of life because with voluntary consumption I would expect differences in the first weeks of life of piglets. In my experience with voluntary consumption of iron, there is a usually high variability among piglets in the first weeks of life. So take this only as a recommendation for future studies.

We agree that it would have been very interesting to make measures at two stages, one around 2-3 weeks of age and one around weaning (6 weeks in organic pig production) but it was not possible in such a on-farm experiment where access to the piglets is difficult, especially in outdoor farms. However, we have performed a subsequent experiment in an experimental organic farm and performed measures at several time points during lactation as you recommend. Present data show that around 6 weeks of age, variations due to iron supply by neonatal injection, indoors, or due to the housing system are still noticeable. Therefore, it was a “sensitive” stage.

# Response

**Published:** 2022-03-11

**Authors:** armelle prunier

**Affiliations:** inrae, France

Thanks a lot for your valuable evaluation and comments.

- *Please add information about the creep feet I mean iron content and from which day it was offered to the piglets.*

It is an on-farm experiment and we do not have precise information on that. We have added a table (Table 1) to describe all the information we got during the farm visits together with some other characteristics of the farms as requested in other comments. We also added some comments in the text.

- *Please add information about the weaning age of piglets.*

Age at sampling and days from weaning were included in the text. Therefore, age at weaning could be deduced from that. However, we have added this information in Table 1 and added some comments in the text.

- *Please give information about the breeds of pigs in different farms*.

We have added this information in Table 1 and added some comments in the text.

- *I think that the limitation of the study is that the blood was taken only at the weaning and not for example in the first two weeks of life because with voluntary consumption I would expect differences in the first weeks of life of piglets. In my experience with voluntary consumption of iron, there is a usually high variability among piglets in the first weeks of life. So take this only as a recommendation for future studies.*

We agree that it would have been very interesting to make measures at two stages, one around 2-3 weeks of age and one around weaning (6 weeks in organic pig production) but it was not possible in such an on-farm experiment where access to the piglets is difficult, especially in outdoor farms. However, we have performed a subsequent experiment in an experimental organic farm and performed measures at several time points during lactation as you recommend. Present data show that around 6 weeks of age, variations due to iron supply by neonatal injection, indoors, or due to the housing system are still noticeable. Therefore, it was a “sensitive” stage.

# Peer review: Reviewer response for version 1

**DOI:** 10.21956/openreseurope.15505.r28449

**Published:** 2022-02-04

**Authors:** Glen Almond

**Affiliations:** 1 Department of Population Health & Pathobiology, College of Veterinary Medicine, North Carolina State University (NCSU), Raleigh, NC, USA

**General Summary**

 The research evaluated the various blood parameters in piglets at approximately 42 days of age. Eleven outdoor farms and 10 indoor farms were included in the study. Iron supplementation varied among the indoor farms and only one outdoor farm injected piglets with iron. The indoor pigs had lower haemoglobin concentrations and red blood cell volume. Plasma haptoglobin concentrations were lower in the outdoor pigs than in the indoor pigs. The authors indicated that the piglets from primiparous sows had lower haemoglobin concentrations than pigs from multiparous sows. The authors concluded that iron supplementation is not required for piglets raised outdoors, provided that iron was bioavailable in the soils.

**Specific Comments**

 Page 1:

- Background section – change the word “stock” to sow.
-  Some basic demographics, such as the number of sows/farm, of the 21 farms would be helpful.
- Blood samples were collected at 42 days of age and 11.9 kg live weight. Within the manuscript, daily weight gain is given and analyzed. At any time, were the pigs weighed prior to the end of the study? How was the DWG determined? In the results section, the DWG did not differ – this is confusing without starting body weights.
- What was the definition of anemia? Is 105 g/l indicative of anemia? This definition needs to be defined or a suitable reference included.
- What was the health status of the farms? And the preweaning mortality of the pigs on the 21 farms.
- It is concluded that the outdoor pigs do not require supplementation and that the outdoor pigs gain iron from the soil. At any time or for any farm, were soil samples collected to be analyzed for iron content? What are the bioavailabilities of the insoluble ferric oxides in soil? Would this be dependent on the soil pH? This comment also applies to the discussion on page 8.

Page 3:

- Provide the reference for the certifying bodies that consider iron administration as a drug treatment.
- Methods: the allocation of pigs by gender and size is appropriate. The age of pigs was 42 days, but the range was 34 to 57 days. At 57 days, would one expect that the iron injections in the first few days of life really maintain their influence? The influence of iron injections tends to decrease by 28 days of age. At any time, did the pigs receive solid feed, and if so, what was the iron content of the feed (including the sow feed). 
- The SD is given; however in other parts of the manuscript, the SEM is given. Be consistent.

Page 4:

- Lab analyses: plasma or serum? Or both?
- The live weight and DWG (?) did not differ between housing systems but the age differed by 3 days. One could imply that the iron injection was required for the weight gains in the indoor pigs.

Page 5:

- Relationships between (or among parameters). What is meant by “side”? This is confusing.

Page 6:

- Figure 2: Are the titles on the X axis correct? Should they be indoor and outdoor rather than multiparous and primiparous?
- Figures 2 and 3: For these parameters, it appears that there are significant differences between the housing systems; however, are the values in the normal ranges for pigs at this age?
- … the first three components of the multivariate analysis. What are the components? Define them. For Figure 5, which is interesting, it would be valuable to define the statistical components.

Page 7:

- Figure 4: Haematologic ……… parameters?

Page 8:

 Table 1 Legend – check the P values. P<0.5 is not significant.

 Page 9: 

- 2 ^nd^ paragraph. The authors suggest that the indoor pigs are affected with iron deficiency. Although there are differences between indoor and outdoor pigs, are the values for the indoor pigs within published normal ranges?
- Assuming that the outdoor pigs consume iron from the soil, how much is absorbed by the gut? In other words, is gut absorption sufficient? A key reference would be helpful.
- The influence of the 200 mg iron injections typically wanes after weaning at 21-28 days of age. The indoor pigs were much older than the pigs after a 3 or 4 week lactation. The transition to solid nursery diets is helpful for these indoor pigs. Did any of the indoor pig farms (or outdoor farms) provide creep feed or any type of solid feed prior to weaning at 40+ days?

Is the study design appropriate and does the work have academic merit?

Yes

Is the work clearly and accurately presented and does it cite the current literature?

Partly

If applicable, is the statistical analysis and its interpretation appropriate?

I cannot comment. A qualified statistician is required.

Are all the source data underlying the results available to ensure full reproducibility?

Partly

Are the conclusions drawn adequately supported by the results?

Yes

Are sufficient details of methods and analysis provided to allow replication by others?

Partly

Reviewer Expertise:

40 years of basic and applied research in commercial pig production. Current research is directed at evaluating anemia in commercial pig farms. Previous research studies involved UTI's in sows and non-infectious reproductive failure in pigs.

I confirm that I have read this submission and believe that I have an appropriate level of expertise to confirm that it is of an acceptable scientific standard, however I have significant reservations, as outlined above.

# Response

**Published:** 2022-03-11

**Authors:** armelle prunier

**Affiliations:** inrae, France

Thanks a lot for your valuable evaluation and comments. (Reviewer comments in italics)

Page 1

- *Background section – change the word “stock” to sow.*
   Thanks, we have made the change.
- *Some basic demographics, such as the number of sows/farm, of the 21 farms would be helpful.*
   We have added a table (Table 1) to describe this information together with some other characteristics of the farms as requested in all comments. We also added some comments in the text.
- *Blood samples were collected at 42 days of age and 11.9 kg live weight. Within the manuscript, daily weight gain is given and analyzed. At any time, were the pigs weighed prior to the end of the study? How was the DWG determined? In the results section, the DWG did not differ – this is confusing without starting body weights.*
  Sorry, we should have explained how DWG was calculated. Piglets were not weighted at birth. Therefore, we estimated the DWG by the dividing the weight at blood sampling by the age at blood sampling. Therefore, we have added an explanation in the MM part.
- *What was the definition of anemia? Is 105 g/l indicative of anemia? This definition needs to be defined or a suitable reference included.*
  We have modified the second paragraph of the discussion in order to define anaemia.

- *What was the health status of the farms? And the preweaning mortality of the pigs on the 21 farms.*
  This is an on-farm experiment and health status of the farm is a complex concept that was out of the scope of the present study. We did not record the mortality of the piglets during lactation (we know that it is very challenging in outdoor farms since it is very difficult to record the number of live piglets at birth). However, we recorded the average littersize at weaning as indicated by the farmers. This information is given in Table 1 and commented in the text.
- *It is concluded that the outdoor pigs do not require supplementation and that the outdoor pigs gain iron from the soil. At any time or for any farm, were soil samples collected to be analyzed for iron content? What are the bioavailabilities of the insoluble ferric oxides in soil? Would this be dependent on the soil pH? This comment also applies to the discussion on page 8.*
  That would have been very nice to take soil samples and do measures for bioavailability. We have thought to do it but we would have to solve problems of representative samples of soil in the parks of the sows. Taking into account these problems and the time that was available to perform the samples and measures on animals, collect the information on the farms, we gave up.
Page 3
- *Provide the reference for the certifying bodies that consider iron administration as a drug treatment.*
  In fact, the EU regulation can be interpreted in different ways so that there are differences between countries (and hence certification bodies) and within a certification body between inspectors, at least in France. Therefore, we cannot give a list of certification bodies that consider iron administration as a drug treatment but we have modified the sentence.

- *Methods: the allocation of pigs by gender and size is appropriate. The age of pigs was 42 days, but the range was 34 to 57 days. At 57 days, would one expect that the iron injections in the first few days of life really maintain their influence? The influence of iron injections tends to decrease by 28 days of age.*
  We agree that it would have been very interesting to make measures at an earlier stage when the effects would have been more marked. However, as a first on-farm experiment on organic pigs, we make the choice of describing the situation at a critical moment in piglet life, that is around weaning. In addition, our data show that around 6 weeks of age, variations due to iron supply by neonatal injection, indoors, or due to the housing system are still noticeable.

- *At any time, did the pigs receive solid feed, and if so, what was the iron content of the feed (including the sow feed).*
  It is an on-farm experiment and we do not have precise information on that. We have added all the information we got in the farm visits in Table 1 and in the text.
- *The SD is given; however in other parts of the manuscript, the SEM is given. Be consistent.*
  Regarding the description of the Materials and Methods, we think that the SD is more obvious since the aim is to describe precisely the sample used for the experiment. Regarding the description of the results, we prefer to use the SEM since statistics are based on SEM. It is a common practice but, if really necessary, we can change to SEM all over the text.
Page 4

- *Lab analyses: plasma or serum? Or both?*
  Both were used as indicated in the text “Haptoglobin was analysed on plasma, iron, ferritin, hydroperoxides (= oxidation products = dROM) and the antioxidant capacity of the blood (BAP) on serum.”

- *The live weight and DWG (?) did not differ between housing systems but the age differed by 3 days. One could imply that the iron injection was required for the weight gains in the indoor pigs.*
  Yes, this is probably right. However, to prove it, we should have compared indoor pigs having or not received iron supplementation.
Page 5

- *Relationships between (or among parameters). What is meant by “side”? This is confusing.*
  We have changed the text.
Page 6
- *Figure 2: Are the titles on the X axis correct? Should they be indoor and outdoor rather than multiparous and primiparous?*
  Very sorry for that, it is a mistake (Figure 2 and Figure 4 were the same). When I copied the figures from excel in the format sent to the journal, I made the mistake. Thanks for having pointed out this error. Now, I have sent the right figure comparing indoor and outdoor piglets.
- *Figures 2 and 3: For these parameters, it appears that there are significant differences between the housing systems; however, are the values in the normal ranges for pigs at this age?*
  The aim of the paper was not to determine what are the normal ranges but whether there are differences between housing systems and whether neonatal iron supply is needed. However, we agree that information on what are normal ranges can be very useful. Thanks for pointing that out. Therefore, we have modified the second paragraph of the discussion *.*

- … *the first three components of the multivariate analysis. What are the components? Define them. For Figure 5, which is interesting, it would be valuable to define the statistical components.*
  PCA is a descriptive analysis of the data. Here each piglet has 8 measures that were retained for the analysis. Therefore, the whole set of data (486 piglets) can be represented by an ellipsoid with 8 dimensions. In order to describe this ellipsoid, we make projections on 2 dimensional charts that are the best representations of the ellipsoid. In other words, these charts maximize the dispersion between individuals. They correspond to the first plot using the first and second first principal components, the second plot using the first and third components etc… These components can equivalently be defined as directions that maximize the variance of the projected data. These components are orthogonal. It is not possible to explain the principles of PCA in the text but we have added between brackets.
Page 7

- *Figure 4: Haematologic ……… parameters?*
  Thanks for spotting that. We have added that in the legend.
Page 8
- *Table 1 Legend – check the P values. P<0.5 is not significant.T*T
  Thanks for spotting that. We have changed that in the legend.
Page 9 
- *2 ^nd^ paragraph. The authors suggest that the indoor pigs are affected with iron deficiency. Although there are differences between indoor and outdoor pigs, are the values for the indoor pigs within published normal ranges?*
  We have now changed the second paragraph of the discussion to indicate which values can be considered as normal.

- *Assuming that the outdoor pigs consume iron from the soil, how much is absorbed by the gut? In other words, is gut absorption sufficient? A key reference would be helpful.*
  We do not know how much soil and iron from soil can be absorbed by the gut of the piglets. We only know that high amount can be depicted in the gut and refer to the paper from Venn et al (1947) to illustrate that. However, our observations clearly show that Hb levels were relatively high in outdoor pigs without iron injection. Therefore, iron to synthetize Hb must come from somewhere and the best hypothesis is soil.

- *The influence of the 200 mg iron injections typically wanes after weaning at 21-28 days of age. The indoor pigs were much older than the pigs after a 3 or 4 week lactation. The transition to solid nursery diets is helpful for these indoor pigs. Did any of the indoor pig farms (or outdoor farms) provide creep feed or any type of solid feed prior to weaning at 40+ days?*
  We do not have precise information on the feed consumed by the piglets and from when. However, we have added all the information we got during the farm visits in Table 1 and some comments in the text.
